# Supplementary material for: Modeling glioblastoma heterogeneity as a dynamic network of cell states
Source: Mol Syst Biol. 2021 Sep 16;17(9):e10105. doi: 10.15252/msb.202010105 (PMC8444284; doi:10.15252/msb.202010105)
Supplement: Supplementary file 6 — Source Data for Figure 5 [file MSB-17-e10105-s004.zip › Figure5A_sourcedata/GSEA_3071/nosigdb_stateII.GseaPreranked.1622795151171/SEURAT_CELLCYCLE.html]

Details for gene set SEURAT\_CELLCYCLE[GSEA]

|  || Dataset | state23071 |
| Phenotype | NoPhenotypeAvailable |
| Upregulated in class | na\_pos |
| GeneSet | SEURAT\_CELLCYCLE |
| Enrichment Score (ES) | 0.78901565 |
| Normalized Enrichment Score (NES) | 4.5460362 |
| Nominal p-value | 0.0 |
| FDR q-value | 0.0 |
| FWER p-Value | 0.0 |
Table: GSEA Results Summary

  

Fig 1: Enrichment plot: SEURAT\_CELLCYCLE      
 Profile of the Running ES Score & Positions of GeneSet Members on the Rank Ordered List

  

| PROBE | GENE SYMBOL | GENE\_TITLE | RANK IN GENE LIST | RANK METRIC SCORE | RUNNING ES | CORE ENRICHMENT || 1 | CLSPN |  |  | 1 | 1.984 | 0.0237 | Yes |
| 2 | RRM2 |  |  | 3 | 1.857 | 0.0459 | Yes |
| 3 | ANLN |  |  | 4 | 1.760 | 0.0671 | Yes |
| 4 | TYMS |  |  | 8 | 1.665 | 0.0865 | Yes |
| 5 | CDC6 |  |  | 11 | 1.631 | 0.1057 | Yes |
| 6 | CDK1 |  |  | 14 | 1.540 | 0.1237 | Yes |
| 7 | HJURP |  |  | 17 | 1.490 | 0.1412 | Yes |
| 8 | FEN1 |  |  | 19 | 1.464 | 0.1587 | Yes |
| 9 | UBE2C |  |  | 23 | 1.438 | 0.1753 | Yes |
| 10 | RAD51AP1 |  |  | 25 | 1.384 | 0.1917 | Yes |
| 11 | USP1 |  |  | 31 | 1.357 | 0.2069 | Yes |
| 12 | DTL |  |  | 33 | 1.330 | 0.2227 | Yes |
| 13 | RRM1 |  |  | 37 | 1.310 | 0.2377 | Yes |
| 14 | GINS2 |  |  | 40 | 1.306 | 0.2530 | Yes |
| 15 | AURKB |  |  | 44 | 1.276 | 0.2677 | Yes |
| 16 | HELLS |  |  | 46 | 1.259 | 0.2826 | Yes |
| 17 | PCNA |  |  | 58 | 1.214 | 0.2945 | Yes |
| 18 | CDC45 |  |  | 59 | 1.212 | 0.3092 | Yes |
| 19 | ATAD2 |  |  | 63 | 1.189 | 0.3228 | Yes |
| 20 | PRIM1 |  |  | 65 | 1.170 | 0.3366 | Yes |
| 21 | MSH2 |  |  | 66 | 1.163 | 0.3507 | Yes |
| 22 | POLD3 |  |  | 70 | 1.143 | 0.3637 | Yes |
| 23 | TOP2A |  |  | 72 | 1.132 | 0.3772 | Yes |
| 24 | KIF2C |  |  | 73 | 1.132 | 0.3908 | Yes |
| 25 | MCM5 |  |  | 74 | 1.128 | 0.4044 | Yes |
| 26 | CCNE2 |  |  | 76 | 1.121 | 0.4177 | Yes |
| 27 | UHRF1 |  |  | 79 | 1.113 | 0.4307 | Yes |
| 28 | MCM4 |  |  | 81 | 1.110 | 0.4438 | Yes |
| 29 | GMNN |  |  | 83 | 1.108 | 0.4570 | Yes |
| 30 | NDC80 |  |  | 94 | 1.074 | 0.4674 | Yes |
| 31 | TMPO |  |  | 99 | 1.056 | 0.4792 | Yes |
| 32 | MKI67 |  |  | 107 | 1.031 | 0.4899 | Yes |
| 33 | BRIP1 |  |  | 110 | 1.029 | 0.5018 | Yes |
| 34 | TPX2 |  |  | 111 | 1.025 | 0.5142 | Yes |
| 35 | RFC2 |  |  | 112 | 1.022 | 0.5265 | Yes |
| 36 | CDCA8 |  |  | 113 | 1.018 | 0.5388 | Yes |
| 37 | KIF11 |  |  | 115 | 1.014 | 0.5508 | Yes |
| 38 | NUF2 |  |  | 125 | 1.004 | 0.5607 | Yes |
| 39 | CKS1B |  |  | 128 | 0.999 | 0.5722 | Yes |
| 40 | TACC3 |  |  | 130 | 0.992 | 0.5840 | Yes |
| 41 | KIF20B |  |  | 133 | 0.989 | 0.5954 | Yes |
| 42 | WDR76 |  |  | 142 | 0.975 | 0.6052 | Yes |
| 43 | EXO1 |  |  | 145 | 0.969 | 0.6164 | Yes |
| 44 | CKAP2L |  |  | 148 | 0.959 | 0.6274 | Yes |
| 45 | DSCC1 |  |  | 155 | 0.946 | 0.6374 | Yes |
| 46 | BLM |  |  | 156 | 0.942 | 0.6487 | Yes |
| 47 | KIF23 |  |  | 161 | 0.936 | 0.6590 | Yes |
| 48 | GTSE1 |  |  | 167 | 0.929 | 0.6690 | Yes |
| 49 | RPA2 |  |  | 169 | 0.929 | 0.6800 | Yes |
| 50 | CDCA2 |  |  | 171 | 0.922 | 0.6909 | Yes |
| 51 | MCM6 |  |  | 176 | 0.906 | 0.7008 | Yes |
| 52 | CDCA3 |  |  | 219 | 0.858 | 0.7007 | Yes |
| 53 | CHAF1B |  |  | 229 | 0.845 | 0.7086 | Yes |
| 54 | TUBB4B |  |  | 271 | 0.784 | 0.7079 | Yes |
| 55 | HMGB2 |  |  | 276 | 0.780 | 0.7163 | Yes |
| 56 | SMC4 |  |  | 279 | 0.777 | 0.7252 | Yes |
| 57 | CENPE |  |  | 284 | 0.775 | 0.7335 | Yes |
| 58 | CENPA |  |  | 303 | 0.751 | 0.7381 | Yes |
| 59 | CKS2 |  |  | 312 | 0.745 | 0.7451 | Yes |
| 60 | SLBP |  |  | 318 | 0.740 | 0.7528 | Yes |
| 61 | TIPIN |  |  | 326 | 0.732 | 0.7599 | Yes |
| 62 | AURKA |  |  | 360 | 0.705 | 0.7602 | Yes |
| 63 | TTK |  |  | 365 | 0.701 | 0.7676 | Yes |
| 64 | E2F8 |  |  | 379 | 0.691 | 0.7727 | Yes |
| 65 | POLA1 |  |  | 443 | 0.654 | 0.7649 | Yes |
| 66 | NCAPD2 |  |  | 457 | 0.647 | 0.7695 | Yes |
| 67 | HMMR |  |  | 475 | 0.639 | 0.7730 | Yes |
| 68 | BIRC5 |  |  | 505 | 0.628 | 0.7733 | Yes |
| 69 | RAD51 |  |  | 506 | 0.627 | 0.7809 | Yes |
| 70 | ECT2 |  |  | 510 | 0.626 | 0.7877 | Yes |
| 71 | CENPF |  |  | 575 | 0.588 | 0.7788 | Yes |
| 72 | CKAP2 |  |  | 619 | 0.569 | 0.7750 | Yes |
| 73 | CDC25C |  |  | 626 | 0.568 | 0.7804 | Yes |
| 74 | NUSAP1 |  |  | 654 | 0.550 | 0.7803 | Yes |
| 75 | DLGAP5 |  |  | 712 | 0.529 | 0.7724 | Yes |
| 76 | MCM2 |  |  | 734 | 0.521 | 0.7735 | Yes |
| 77 | RANGAP1 |  |  | 736 | 0.520 | 0.7795 | Yes |
| 78 | NASP |  |  | 741 | 0.518 | 0.7848 | Yes |
| 79 | UNG |  |  | 750 | 0.516 | 0.7890 | Yes |
| 80 | CDC20 |  |  | 844 | 0.486 | 0.7717 | No |
| 81 | UBR7 |  |  | 857 | 0.483 | 0.7745 | No |
| 82 | CASP8AP2 |  |  | 1025 | 0.436 | 0.7382 | No |
| 83 | NEK2 |  |  | 1100 | 0.417 | 0.7248 | No |
| 84 | CBX5 |  |  | 1132 | 0.412 | 0.7220 | No |
| 85 | BUB1 |  |  | 1145 | 0.408 | 0.7239 | No |
| 86 | ANP32E |  |  | 1334 | 0.371 | 0.6815 | No |
| 87 | CKAP5 |  |  | 2135 | 0.258 | 0.4852 | No |
| 88 | GAS2L3 |  |  | 2755 | -0.327 | 0.3348 | No |
Table: GSEA details [plain text format]

  

Fig 2: SEURAT\_CELLCYCLE: Random ES distribution      
 Gene set null distribution of ES for **SEURAT\_CELLCYCLE**
